# Chemometric‐Assisted Extraction Optimization and HPLC‐MS Profiling of *Mentha* × *villosa* Targeting Photoprotective Activity

**DOI:** 10.1002/cbdv.71764

**Published:** 2026-10-09

**Authors:** Juliana de Medeiros Gomes, Thalisson Amorim de Souza, Márcio Vinícius Cahino Terto, Maria Beatriz Félix Mendes Nunes, Hariel Maffei Umesaki Tessaro, Joanda Paolla Raimundo e Silva, Samuel Paulo Cibulski, Luiz Alberto Lira Soares, Vera Isaac, Marcelo Sobral da Silva, Josean Fechine Tavares

**Affiliations:** ^1^ Laboratório Multiusuário de Caracterização e Análise (LMCA) Universidade Federal da Paraíba João Pessoa Paraíba Brazil; ^2^ Laboratório De Cosmetologia, Faculdade De Ciências Farmacêuticas Universidade Estadual Paulista Araraquara São Paulo Brazil; ^3^ Departamento De Farmácia Universidade Estadual de Maringá Maringá Paraná Brazil; ^4^ Faculdade De Ciências da Saúde do Trairi Universidade Federal do Rio Grande Do Norte Santa Cruz Rio Grande do Norte Brazil; ^5^ Núcleo De Desenvolvimento Analítico e Tecnológico de Fitoterápicos Universidade Federal de Pernambuco Recife Pernambuco Brazil

**Keywords:** factorial design, Lamiaceae, mojito mint, natural products

## Abstract

*Mentha × villosa* is a food and medicinal plant with long history of use. However, its use as potential ingredient for cosmetic formulations continues underexplored. Based on that, the present study sought to optimize the extraction of *M. × villosa* (MV‐OE), characterize its composition, evaluate its antioxidant capacity and photoprotective effects alone and in combination with ethylhexyl methoxycinnamate (EHMC), a commercial sunscreen filter, in prototype formulations. Extraction was optimized through DoE, total phenolic content, and rosmarinic acid were quantified, while antioxidant activity was determined by DPPH and ABTS assays. The chemical profile of extracts was analyzed by HPLC‐DAD‐ESI‐MS^n^ and by HPLC‐ESI‐HRMS, supported by PCA. The sun protection factor (SPF) was determined using an in vitro UV‐transmission method. MV‐OE showed significant phenolic content and antioxidant activity, PCA proved effective for discriminating the extracts according to their phenolic composition. Besides, dereplication led to tentative assignment of 30 compounds. Formulation prototypes 5 and 6 achieved significant SPF values and enhanced EHMC performance. Altogether, these findings highlight MV‐OE as a potential ingredient for future sunscreen formulations.

## Graphical abstract

Design‐of‐experiments optimization, followed by LC–MS profiling and chemometric analysis, led to a phenolic‐rich *Mentha* × *villosa* extract with strong antioxidant activity and photoprotective effects enhancing the performance of ethylhexyl methoxycinnamate in sunscreen‐based formulations.

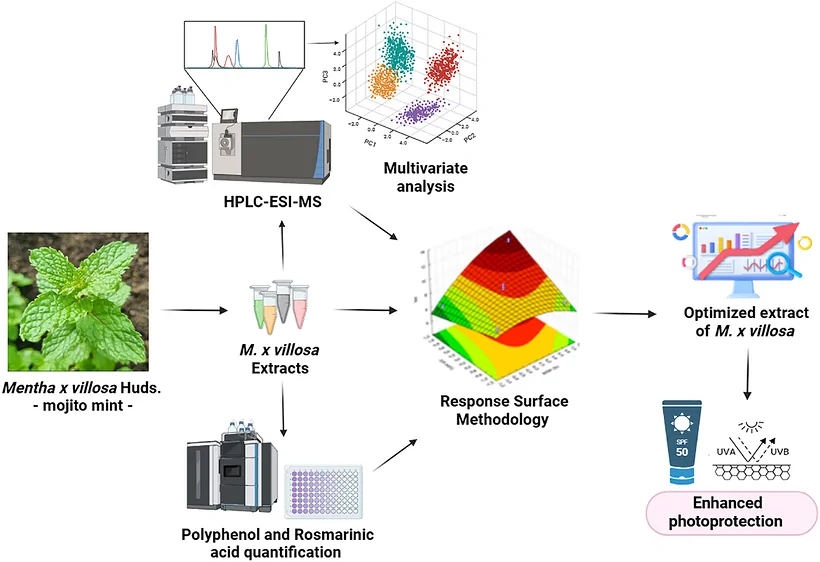

## 1 Introduction

Recent years have witnessed a significant rise in the use of natural compounds as active agents in the development of cosmetic products. Among their benefits over the synthetics, they show broad skin‐type compatibility and reduce likelihood of side effects. Hence, medicinal plants have served as a crucial source and driving force for the cosmetic industry [1]. The ultraviolet radiation, mainly UVA and UVB (wavelength ranges 315–400 nm and 280–315 nm, respectively), is harmful to human skin, excessive exposure may lead to several acute effects, mainly sunburn, and skin tanning, as well as chronic outcomes. The principal chronic effects include cellular and degenerative changes, photoaging, and skin cancer [2]. Considering these deleterious effects, innovative strategies, and more efficient products, especially sunscreens formulations, have become a current need in modern society.

In this context, phenolic compounds such as stilbenes, flavonoids and their acid derivatives are particularly noteworthy. These natural antioxidants also provide high protection against both types of radiation through different mechanisms, inducing collagen and elastin synthesis, directly absorbing UV radiation, inhibition of collagenases and elastases and even activating repair mechanisms of DNA [3]. Species rich in such compounds may offer significant materials for the development of photoprotective formulations [4]. Given the remarkable potential of natural products for sunscreens development, *Mentha × villosa* (Lamiaceae), popularly known as mojito mint, has displayed significant biological activities, particularly antioxidant effects. These properties are attributed to their diverse chemical composition, marked by high amounts of rosmarinic acid (RA) and flavonoids [5].

The yield and composition of secondary metabolites obtained from plant extracts are influenced by several factors, including extraction method, solvent, plant part, and drug‐to‐solvent ratio. Assessing these variables has become essential to ensure reproducibility and quality in plant derived products. Factorial designs and surface response methods provide effective approaches to examine the individual and combined effects of these parameters while minimizing resource consumption [6]. In addition, advances in analytical methodologies enable precise compound characterization, facilitating the identification and quantification of constituents in plant extracts [7]. Integrating these techniques offers valuable insights into extract composition and support the standardization for biological screening and assays.

Based on these considerations, the present study aimed to optimize the extraction of *Mentha × villosa* using 2^2^ factorial design, evaluate the chemical profile of the different extraction conditions, annotate the main compounds by HPLC‐MS experiments, evaluate their antioxidant effects, and photoprotective potential alone and in combination with the solar filter ethylhexyl methoxycinnamate (EHMC), incorporated into a commercial self‐emulsifying base, herein referred as prototype formulations once further studies are required for product development.

## 2 Results and Discussion

### 2.1 Extraction Optimization of *M*. × *Villosa*: RA and TPC Content, Dry Residue (DR) and Extraction Efficiency (EE)

Phenolic compounds and flavonoids play a pivotal role in the photoprotective activity of *M. × villosa* extract. A previous study of seasonality clearly demonstrates the correlation between the activity and the concentration of these secondary metabolites in *M. × villosa* [8] To optimize their extraction conditions, a 2^2^ factorial design with central points was applied targeting polyphenols and rosmarinic acid (RA), the chemical marker for this species and main responsible for its antioxidant effect. The conditions were established as follows; ethanol concentration (50%, 70%, 90% v/v) and plant proportion (2.5%, 5%, 7.5% w/v) were evaluated as independent variables. The resulting extracts exhibited marked variation in rosmarinic acid (RA), total polyphenol content (TPC), dry residue (DR), and extractive efficiency (EE). Table 1 represents the matrix and the respective variables for each parameter.

**TABLE 1:** Matrix of factorial design 2^2^ with central points.

Exp	Codified variables		Natural variables		RA (g%)	TPC (g%)	DR (g%)	EE
	EtOH (%)	P:S (%; m/v)	EtOH (%)	P:S (%; m/v)				
1	−1	−1	50	2.5	8.04	28.58	0.96	29.82
2	1	−1	90	2.5	7.42	23.42	0.68	34.57
3	−1	1	50	7.5	6.27	28.31	2.75	10.31
4	1	1	90	7.5	11.79	20.66	1.86	11.09
5	0	0	70	5	9.56	27.44	1.70	16.15
6	0	0	70	5	9.21	27.46	1.79	15.33
7	0	0	70	5	9.14	27.28	1.83	14.89

abbreviations: DR, dry residue; EE, extractive efficiency; Exp, experiment; P:S, powder/solvent ratio; RA, rosmarinic acid; TPC, total polyphenols content.

The mathematical model obtained demonstrated a good coefficient of multiple correlations (*R* ^2^). For TPC and EE correlation, the coefficients indicated over 99% of the experimental variance, explained by the Equations 1 and 2. The model was still able to explain 97% and 98% of the variances found for RA content and DR, Equations (3) and (4), respectively. The equations and results are herein summarized.

(1) TPC=27.39−3.20x1−1.18x2−2.15x1x2−0.92

(2) EE=15.45+1.38x1−10.75x2+5.98x1x2−0.99

(3) RA=9.30+1.23x1+0.63x2+1.54x1x2−0.93

(4) DR=1.78−0.29x1+0.74x2−0.21x1x2−0.15

Linear terms as well as their interactions showed great influences for both factors, ethanol content and plant proportion (powder:solvent ratio). Hence, they also impacted over the responses studied, as demonstrated by the surface plots (Figure 1).

**FIGURE 1:**
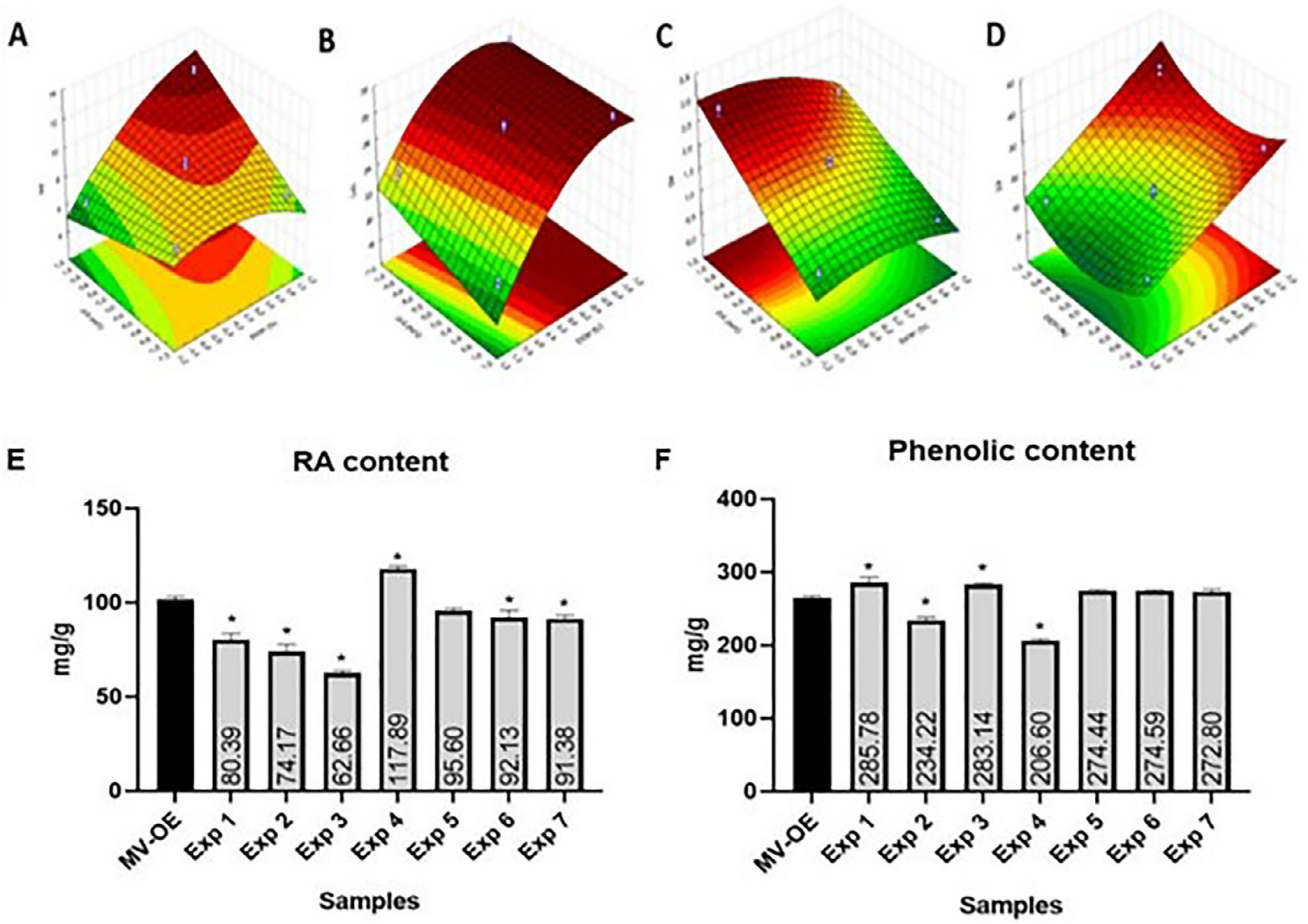
Response surface plots illustrating the effects of ethanol concentration and plant proportion on the evaluated parameters: (A) rosmarinic acid, (B) total polyphenols, (C) dry residue content, and (D) extraction efficiency. Bar graphs show (E) rosmarinic acid content and (F) total polyphenol content. Asterisks (*) indicate statistically significant differences compared to MV‐OE (*p* < 0.05).

Ethanol concentration and plant proportion significantly influenced extraction outcomes, both individually and through interaction effects. Higher ethanol percentages combined with lower plant proportion favored RA extraction (Figure 1A,E), while TPC was primarily driven by ethanol concentration (Figure 1B,F). Dry residue increased proportionally with plant proportion, whereas EE was more sensitive to the powder‐to‐solvent ratio (Figure 1D). Response surface and contour plots confirmed these trends and guided the selection of optimal conditions. The desirability function indicated that extraction with 70% ethanol and 5% plant proportion provided the best balance among all responses, pareto charts, and contour plots for each parameter are available as Supporting Information (Figures S1–S9, l). Under these conditions, the optimized extract (MV‐OE) exhibited high phenolic content (274.58 ± 2.83 mg GAE/g) and elevated RA levels (101.96 ± 1.26 mg/g), surpassing values previously reported for other *Mentha* species, under comparable or more exhaustive extraction conditions [9, 10, 11, 12]. These findings reinforce the relevance of statistical optimization for producing enriched extracts suitable for industrial applications

### 2.2 DPPH and ABTS Radicals Scavenging Activity

Antioxidant activity is also beneficial for development of skin products, including sunscreen formulations. After optimization, antioxidant activity of MV‐OE was evaluated by two radicals scavenging assays, DPPH and ABTS. For the DPPH, MV‐OE exhibited an IC_50_ = 42.44 ± 0.28 µg/mL, compared to a previous analysis of *M. × villosa* extracts, throughout 12 months, the best result was an IC_50_ at 75.09 µg/mL [8]. These differences reveal that optimization significantly increased the DPPH scavenging capacity. Furthermore, MV‐OE also demonstrated a better IC_50_ than those found in other *Mentha* species reported in literature. Ranjbar et al., 2020 tested four species of *Mentha*, with the best IC_50_ at 60.9 µg/mL for June‐harvested *M. x piperita var. citrate* [13]. In another study, nine *Mentha* species were tested, only horse mint (*M. longifolia*) ethanolic extract had comparable DPPH scavenging to MV‐OE [14]. Nickavar et al., 2008 reported an IC_50_ of 87.89 µg/mL for *M. spicata*, which is closely related to mojito mint species [15]. For ABTS radical scavenging, MV‐OE had an IC_50_ of 322.87 ± 5.28 µg/mL, higher than the 173.80 µg/mL seen for *M. spicata* [16]. Additional studies also found stronger ABTS scavenging for some *Mentha* extracts than MV‐OE [17].

### 2.3 HPLC‐MS: Principal Component Analysis (PCA) and Chemical Profile

To shed light on a detailed chemical composition of *M. × villosa* hydro alcoholic extracts, HPLC‐MS experiments were carried out for all samples. Initially, HPLC‐ESI‐HRMS data was used to generate a PCA plot. The results revealed a discrimination pattern for experiments 1, 2, and 4 (Figure 2).

**FIGURE 2:**
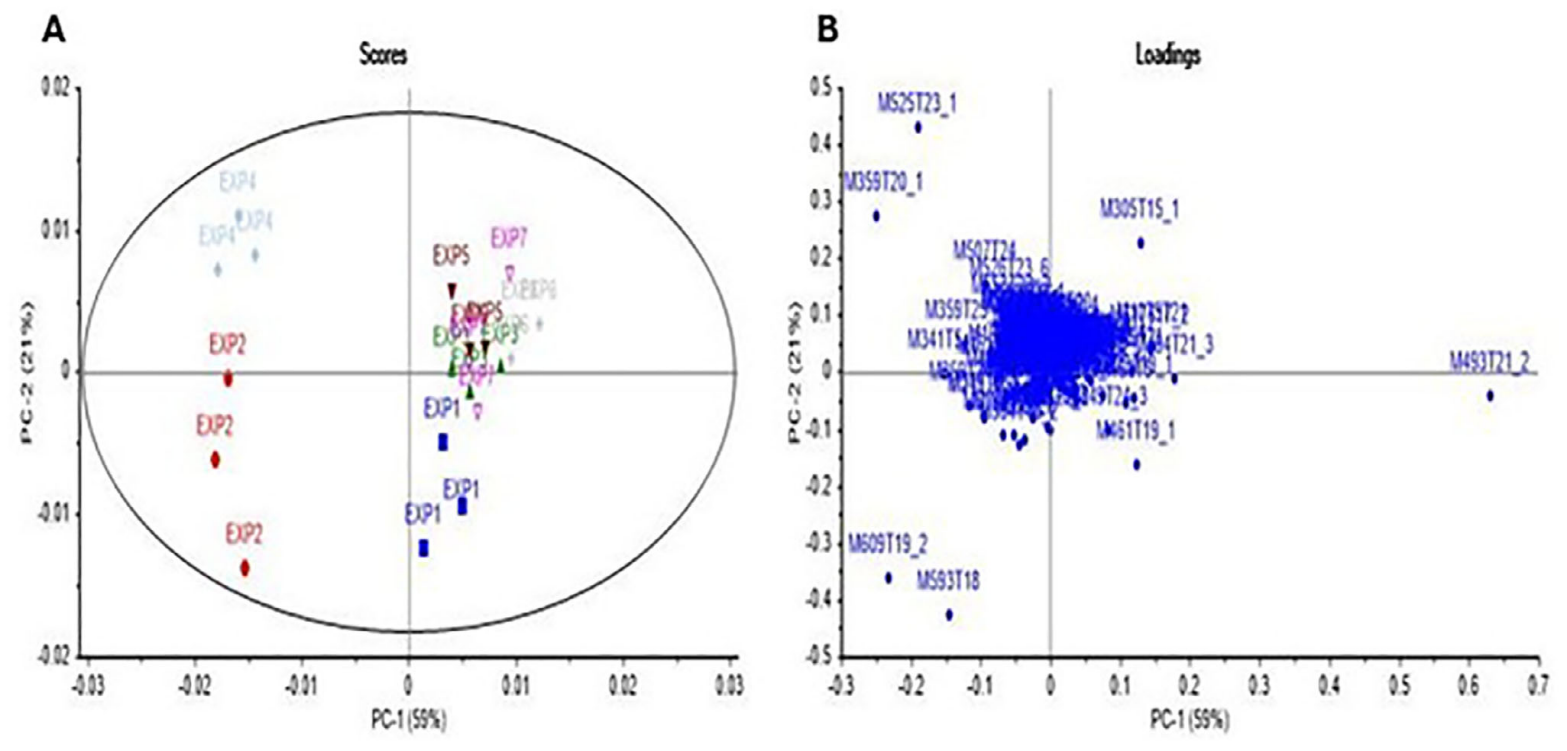
PCA scores plot (A) and loadings plot (B) (PC1 vs. PC2) of experiments 1 to 7 analyzed by HPLC‐HRMS.

The score plot (Figure 2A), PC1 explained 59% and PC2 21% of variance, summing to 80% total explained variance. The grouping pattern suggests a clear distribution based on the phenolic content (Figure 1F), the samples with small amounts grouped in the left, meanwhile those with higher content grouped in the right. These findings also point out the reproducibility of extraction procedures, especially for Exp 3,6,5 and 7. At the right side, the loadings plot (Figure 2B) shows in evidence the ions attributed to *m/z* 525.1780, 359.0776, 609.1841, 593.1521, 305.0708, 461.0742, and 493.1150. These seven features represent the main contributors associated with the discrimination pattern of the extracts; each feature was tentatively associated with a natural metabolite. To tentatively assign their identities, tandem mass spectrometry experiments were also carried out. As result, thirty substances were proposed by their retention time, fragmentation patterns and exact molecular mass, as summarized in Table 2.

**TABLE 2:** Tentatively identified compounds from *M. × villosa* extract by HPLC‐ESI‐DAD‐MS^n^.

Peak	R.T.	*m/z* [M−H]^−^	Molecular formula	Error (ppm)	MS^n^ *m/z*	Compound	Reference
1	5.5	191.0558	C_7_H_12_O_6_	1.6	MS^2^ [191]: 173, 127, 93, 85	Quinic acid	[18]
2	20.0	315.0715	C_13_H_16_O_9_	2.1	MS^2^ [315]: 153 MS^3^ [315→153]: 109	Protocatechuic acid hexoside	[19]
3	20.5	197.0453	C_9_H_10_O_5_	1.3	MS^2^ [197]: 179 MS^3^ [197→179]: 135	Salvianic acid A	[20]
4	23.7	353.0872	C_16_H_18_O_9_	1.8	MS^2^ [353]: 191, 179,135 MS^3^ [353→191]: 173, 127,111, 93, 85	3‐caffeoylquinic acid	[21]
5	25.6	359.0967	C_15_H_20_O_10_	4.6	EM^2^ [359]: 197 MS^3^ [359→197]: 182, 153, 138	Syringic acid hexoside	[22]
6	32.6	353.0885	C_16_H_18_O_9_	−2.0	MS^2^ [353]: 191, 179, 173,135 MS^3^ [353→173]: 111, 93	4‐caffeoylquinic acid	[23]
7	35.2	179.0356	C_9_H_8_O_4_	−3.3	MS^2^ [179]: 135	Caffeic acid	[24]
8	35.4	387.1672	C_18_H_28_O_9_	−2,8	MS^2^ [387]: 369, 225, 207, 163	Tuberonic acid hexoside	[25]
9	35.8	305.0708	C_12_H_18_O_7_S	−2.4	MS^2^ [305]: 225	12‐hydroxy jasmonate sulfate	[26]
10	37.9	593.1528	C_27_H_30_O_15_	−2.8	MS^2^ [593]: 575, 503, 473, 413, 383, 353	Vicenin‐2	[27]
11	39.4	537.1054	C_27_H_22_O_12_	−2.9	MS^2^ [537]: 493, 313, 295 MS^3^ [537→493]: 313, 295	Lithospermic acid I	[28]
12	42.4	595.1674	C_27_H_32_O_15_	−1.0	MS^2^ [595]: 287 MS^3^ [595 → 287]: 269, 151, 135, 125, 107	Eriodictyol rutinoside	[29]
13	43.1	717.1466	C_36_H_30_O_16_	−0,7	MS^2^ [717]: 537, 519, 339, 321 MS^3^ [717→519]: 339, 321	Salvianolic acid E	[30]
14	44.8	521.1291	C_24_H_25_O_13_	1.9	MS^3^ [521]: 359 MS^2^ [521→359]: 223, 197, 179, 161	Rosmarinic acid hexoside	[31]
15	46.9	579.1731	C_27_H_32_O_14_	−2.1	EM^2^[579]: 271 MS^3^ [579→271]: 177, 151	Narirutin	[32]
16	47.2	593.1521	C_27_H_30_O_15_	−1.5	MS^2^ [593]: 285 MS^3^ [593→285]: 267, 257, 243, 241, 213, 217, 197, 199, 175, 151, 133	Luteolin rutinoside	[20]
17	48.0	447.0940	C_21_H_20_O_11_	−1.6	MS^2^ [447]: 285 MS^3^ [447→285]: 267, 257, 243, 241, 213, 217, 197, 199, 175, 151, 133	Luteolin hexoside	[33]
18	48.5	461.0742	C_21_H_18_O_12_	−3.7	MS^2^ [461]: 285 MS^3^ [461→285]: 267, 257, 243, 241, 213, 217, 197, 199, 175, 151, 133	Luteolin glucuronide I	[34]
19	48.9	609.1841	C_28_H_34_O_15_	−2,6	MS^2^ [609]: 301 MS^3^ [609→301]: 286, 283, 258, 257, 151, 125	Hesperidin	[35]
20	49.6	609.1476	C_27_H_30_O_16_	−2.4	MS^2^ [609]: 301 MS^3^ [609→301]: 271, 179, 151	Rutin	[34]
21	51.0	359.0776	C_18_H_16_O_8_	−0.9	MS^2^ [359]: 197, 179, 161	Rosmarinic acid^*^	[36]
22	51.3	537.1046	C_27_H_22_O_12_	−1.3	MS^2^ [537]: 493, 359 MS^3^ [537→493]: 359, 313, 295	Lithospermic acid II	[20]
23	52.0	607.1678	C_28_H_32_O_15_	−1.7	MS^2^ [607]: 299, 284, 256 MS^3^ [607→299]: 284 MS^3^ [607→284]: 256, 151	Diosmin	[29]
24	57.1	461.0734	C_21_H_18_O_12_	−1.8	MS^2^ [461]: 285 MS^3^ [461→285]: 267, 257, 243, 241, 213, 217, 197, 199, 175, 151, 133	Luteolin glucuronide II	[34]
25	57.1	493.1150	C_26_H_22_O_10_	−1.9	MS^2^ [493]: 359, 357, 313 MS^3^ [493→359]: 223, 197, 179, 161	Salvianolic acid A	[37]
26	57.4	593.1871	C_28_H_34_O_14_	0.8	MS^2^ [593]: 285 MS^3^ [593→285]: 270, 257, 243, 241, 228, 226, 217,199, 164, 151, 136, 125, 107	Isosakuranetin rutinoside	[38]
27	62.0	285.0401	C_15_H_10_O_6_	1.4	MS^2^ [285]: 267, 257, 243, 241, 213, 217, 197, 199, 175, 151, 133	Luteolin	[39]
28	64.2	313.0716	C_17_H_14_O_6_	0.5	MS^2^ [313]: 269 MS^3^ [313→269]: 159, 109	Salvianolic acid F	[20]
29	64.5	525.1780	C_28_H_30_O_10_	−2.6	MS^2^ [525]: 341, 359, 161, 179, 221 MS^3^ [525→359]:	Rosmarinic acid derivative	[20]
30	65.0	359.0776	C_18_H_16_O_8_	−0.9	MS^2^ [359]: 344, 329, 313, 179, 161 MS^3^ [359→344]: 329	Jaceidin	[40]

* Confirmed by comparison with standard.

Based on PCA (Figure 2) and dereplication results (Table 2), these main features indicate which compounds were most influenced by the tested conditions and highlight the importance of combining different analytical techniques as tools to ensure the quality of extracts intended for pharmaceutical applications. In this context, 12‐hydroxyjasmonate sulfate (*m/z* 305.0708) and salvianolic acid A (*m/z* 493.1150) contributed to the similar chemical profile observed in extracts from experiments 3, 5, 6, and 7. Besides, salvianolic acid A constitute a notable feature in PC‐1 loading plot, which indicate a strong correlation between this compound and the enriched phenolic samples. A recent study also demonstrates that salvianolic acid A is able mitigate UV‐B induced skin damage by inhibiting cGAS‐STING activation [41]. Regarding the other features, *m/z* 461.0742, attributed to luteolin glucuronide I, was associated with the discrimination of group 1, which showed the highest total phenolic content. Additionally, experiments 2 and 4 differed due to the presence of hesperidin (*m/z* 609.1841), luteolin rutinoside (*m/z* 593.1521), rosmarinic acid (*m/z* 359.0776), and a rosmarinic acid derivative (*m/z* 525.1780), respectively. Among them, group 4 showed better antioxidant activity, explained by increased amounts of rosmarinic acid.

Careful analysis of the chemical composition, together with quantitative and chemometric data, revealed that although experiment 4 maximized the recovery of rosmarinic acid (Figures 1E and 2B), it did not provide the best overall performance. Instead, the desirability function identified the condition that simultaneously optimized total phenolic content, dry residue, and extractive efficiency, suggesting that the combined contribution of minor compounds would favor the intended photoprotective application without significantly compromising antioxidant activity. Based on these findings, the selected conditions were applied on a larger scale to obtain sufficient extract for incorporation into the self‐emulsifying base and subsequent evaluation of sun protection factor (SPF).

### 2.4 In Vitro Determination of Sun Protection Factor (SPF), UVA Protection Factor (UVA‐PF), and Critical Wavelength

To evaluate the effect of incorporating the optimized extract (MV‐OE) on the photoprotective activity of a potential formulation, eight Polawax‐based prototype formulations were developed containing the extract alone or combined with ethylhexyl methoxycinnamate (EHMC), a commercial UV filter, at different concentrations (as described in the Materials and Methods). The prototype formulations were evaluated using an in vitro UV transmission method, in which the SPF, UVA‐PF, and critical wavelength were determined. The in vitro SPF results revealed that formulation prototypes containing 10% of MV‐OE were able to increase EHMC SPF. Among them, the prototypes encoded as 5 (MV‐OE 10% + EHMC 1%) and 6 (MV‐OE 10% + EHMC 3%) demonstrated the highest values of SPF (23,20, and 30,63, respectively) Table 3.

**TABLE 3:** SPF, UVA‐PF, and critical wavelength of formulation prototypes.

Formulations prototypes	1	2	3	4	5	6	7	8
**SPF**	5.35 ± 0.26	13.15 ± 0.67	17.09 ± 1.58	9.74 ± 2.20	23.20 ± 1.02	30.63 ± 2.10	4.23 ± 0.26	6.27 ± 0.47
**UVA‐PF**	4.27 ± 0.17	6.32 ± 0.39	8.01 ± 0.94	7.85 ± 1.88	12.25 ± 0.26	16.53 ± 1.84	1.65 ± 0.06	2.30 ± 0.18
**Critical wavelength (nm)**	374.10 ± 0.26	368.47 ± 0.32	366.10 ± 0.62	379.67 ± 0.25	374.67 ± 0.30	372.73 ± 0.40	330.23 ± 0.21	333.27 ± 1.62

These findings indicate an additive or synergistic interaction between the natural constituents of MV‐OE and the commercial UV filter. Apart from prototypes 1 and 7, all formulations achieved SPF values above the minimum requirement (SPF ≥ 6) established by the Brazilian Regulatory Agency [42]. Notably, prototypes 1 and 7 contained the lowest concentrations of MV‐OE and EHMC alone, reinforcing the evidence of SPF augmentation. The increase in SPF in sunscreen formulations resulting from the supplementation with phenolic‐rich extracts or isolated compounds, particularly ferulic and rosmarinic acids, and commercial UV filters have been previously reported in the literature [43, 44], supporting the observed effects.

From a structural perspective, ferulic acid, rosmarinic acid, salvianolic acid A, and EHMC share a cinnamic acid‐derived scaffold, which has been extensively associated with photoprotective activity [45]. Their chemical structures are presented in Figure 3. The photoprotective and antioxidant properties of these molecules are associated with several complementary structural features: (i) the conjugated vinyl bridge linking the aromatic ring to the carboxyl group, which extends electron delocalization, enhances UV absorption, and improves molecular stability; (ii) methoxy substituents on the aromatic ring, which promote intramolecular hydrogen bonding and increase resistance to photodegradation; (iii) phenolic hydroxyl groups, which readily donate hydrogen atoms or electrons to quench reactive oxygen species generated upon UV exposure; and (iv) the carboxyl group, which contributes to limiting lipid peroxidation and helps preserve membrane integrity under oxidative stress [46]. These structural characteristics explain the remarkable photoprotective performance of cinnamic acid derivatives and support the enhanced activity observed for the MV‐OE containing formulations.

**FIGURE 3:**
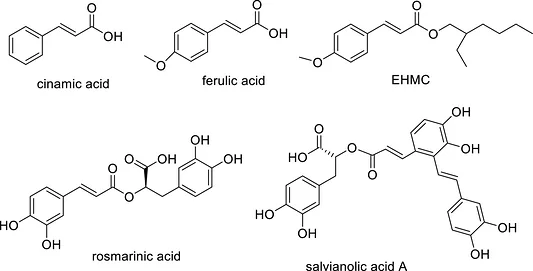
Cinnamoyl‐containing compounds associated with antioxidant activity and photoprotection: ferulic acid, ethylhexyl methoxycinnamate (EHMC), rosmarinic acid, and salvianolic acid A.

In addition to SPF, the evaluation of the UVA protection factor (UVA‐PF) is essential for assessing the overall photoprotective performance of sunscreen formulations. The UVA region is further divided into UVA‐II (320–340 nm) and UVA‐I (340–400 nm), and regulatory agencies worldwide require the UVA‐PF to correspond to at least one‐third of the SPF value [42, 47, 48]. Accordingly, the formulations developed in this study also exhibited promising UVA‐PF values, particularly prototypes 5 and 6, which reached UVA‐PF values of 12.25 and 16.53, respectively. Furthermore, these formulations presented critical wavelengths of 374.67 and 372.73 nm, indicating their potential to provide broad‐spectrum UV protection. In this context, it is worth nothing that the development of formulations capable of extending protection throughout the entire UVA range, particularly within the UVA‐I region (340–400 nm), remains a major challenge in sunscreen research. Unlike UVB radiation, UVA‐I penetrates deeply into the skin, reaching both the epidermis and dermis, where it induces oxidative stress through the excessive generation of reactive oxygen species (ROS). This process promotes lipid peroxidation, protein oxidation, and DNA photodamage, ultimately contributing to photoaging and carcinogenesis [49, 50]. Therefore, formulations capable of maintaining high SPF while providing effective UVA protection, especially combining UV filters and natural photoprotective compounds, represent a promising strategy for the development of safer and more efficient broad‐spectrum sunscreen.

Other concerns regarding sunscreen formulations are associated with the potential adverse effects resulting from long‐term exposure to synthetic UV filters. The occurrence of ethylhexyl methoxycinnamate (EHMC), together with other UV filters, has been documented in human breast milk, urine, and marine organisms, including shellfish from the Mediterranean Sea and the Atlantic coast of France. Furthermore, EHMC becomes a substance of concern because of evidence suggesting potential endocrine‐disrupting properties agent [51]. Given this scenario, identifying natural extracts capable of reducing the amount of synthetic UV filters required in sunscreen formulations while maintaining high sun protection performance has become an important strategy to meet current public health and environmental demands [52].

In this context, MV‐OE showed promising performance, as the prototype formulations achieved satisfactory SPF values while containing low concentrations of EHMC (≤3%). Regulatory agencies establish maximum allowable concentrations for individual UV filters rather than prescribing target concentrations for sunscreen formulations. For this reason, the maximum permitted concentration of EHMC ranges from 7.5% to 10%, depending on the jurisdiction. Hence, the concentration adopted in experimental formulations vary considerably across literature. For example, Mota et al. 2020, formulated sunscreens containing 7.5% EHMC, whereas Li et al. 2022 and Macedo et al. 2025 adopted a concentration of 10% [53, 54, 55]. This variability precludes direct comparison of SPF values among studies. Nevertheless, it highlights the importance of considering the proportion of synthetic UV filters rather than SPF values alone. Therefore, the use of MV‐OE in formulations containing only ≤3% EHMC represents a substantial reduction in synthetic UV filter content compared with formulations commonly reported in the literature.

An ideal sunscreen should provide broad‐spectrum protection against both UVA and UVB radiation, as exposure to either wavelength range contributes to skin damage and increases the risk of photoaging and skin cancer. However, before such formulations can reach the market, their efficacy and safety must be thoroughly evaluated using complementary experimental models to confirm their photoprotective performance and minimize the risk of adverse effects associated with topical application. The accessibility, reproducibility, and cost‐effectiveness of in vitro assays justify their widespread use during the early stages of sunscreen development [56]. For this reason, the formulation prototypes developed in this study were initially evaluated using an in vitro UV transmission method. Nevertheless, methodological factors inherent to this technique, including film thickness, and spectral weighting functions, may result in discrepancies between in vitro and in vivo SPF values. Therefore, the photoprotective properties reported herein should be interpreted as preliminary evidence of sunscreen efficacy rather than as a direct prediction of in vivo performance. As perspective, further investigations including standardized in vivo SPF determination, complete UV transmission profile analysis, skin irritation and sensitization studies, and long‐term formulation stability tests, should be conducted to validate the optimized *Mentha × villosa* extract as an active ingredient for sunscreen formulations.

## 3 Conclusions

The combined use of factorial design and response surface methodology proved to be an effective strategy for maximizing the extraction of polyphenols and rosmarinic acid from *Mentha × villosa* leaves. Multivariate analysis and dereplication enabled a detailed chemical characterization of the optimized extract (MV‐OE), supporting its standardization and revealing a rich profile of phenolic acids, flavonoids, and related metabolites. This chemical composition translated into strong antioxidant activity and enhanced photoprotective performance. Notably, formulations containing MV‐OE at 10% and reduced concentrations of ethylhexyl methoxycinnamate (1%–3%) achieved high SPF, UVA‐PF, and critical wavelength values, meeting broad‐spectrum criteria while lowering the amount of chemical UV filter required. These findings highlight *M. × villosa* as a potential renewable source of bioactive compounds useful in the development of sunscreen formulations. Further studies are required to assess MV‐OE in vivo performance. In summary, this work reinforces the broader relevance of natural products, especially those from Lamiaceae species, in cosmetic innovation and open new perspectives for the rational development of sunscreens formulations.

## 4 Experimental Section

### 4.1 Plant Material

*Mentha × villosa* Hudson leaves (1 kg) were collected at the medicinal plant garden of the Institute of Pharmaceuticals Research (7° 8′ 29.875′′ S/34° 50′ 48.757′′ W), Federal University of Paraiba, João Pessoa‐PB, Brazil. A voucher specimen of this species was deposited at the Prisco Bezerra Herbarium of the Federal University of Ceará, Fortaleza‐CE under the number 14.996. The access was also registered at the National System of Genetic Heritage Management and Associated Traditional Knowledge (SISGEN) platform, reference number A3BA60D.

### 4.2 Optimization of Total Polyphenols and RA Extraction of *M. × villosa*

Freshly collected leaves were dried in a circulating‐air oven at 40°C for 72 h. Extraction was performed according to a 2^2^ factorial design with central points. Ethanol concentration (50%, 70%, 90% v/v; cover the polarity range optimal for phenolic extraction) and plant proportion (2.5%, 5%, 7.5% w/v; chosen based on preliminary tests and industrial feasibility) were evaluated as independent variables. Seven extracts were obtained in triplicate. Total polyphenol content, rosmarinic acid (RA), extraction efficiency (EE), and dry residue (DR) were quantified for each extract. The optimized condition was selected based on the desirability function, considering TPC, RA, DR, and EE simultaneously, and designated MV‐OE (optimized *M. × villosa* extract). The optimal condition consisted of 125 g of dried leaves extracted by turbolysis with 70% ethanol (2.5 L), using four 30‐s cycles with 5‐min intervals. Solvent removal was performed under reduced pressure at 40°C, yielding 24.25 g of MV‐OE. The raw matrix of factorial design 2^2^ with central point's presenting all replicates is available as Table S1.

### 4.3 Total Polyphenol Content Quantification

Total polyphenol content (TPC) was determined using the method described by Singleton et al., 1999 [57]. Triplicate 1 mg/ml sample solutions were prepared and 120 µL of each was transferred to eppendorf tubes containing 500 µL of 10% Folin–Ciocalteu reagent. After incubation in the dark for 8 min at room temperature, 400 µL of 7.5% sodium carbonate solution was added to each tube. The tubes were then allowed to stand for 120 min at room temperature. Afterwards, 200 µL of the solutions were transferred to 96‐well plates and the absorbance measured at 765 nm using a microplate reader (H1M, Biotek). Gallic acid was used to construct a standard calibration curve at concentrations of 100, 150, 200, 250, and 300 µg/ml. Results were expressed as mg GAE/g of sample. Figure S10, demonstrates the gallic acid calibration curve used to determine total polyphenols content.

### 4.4 Rosmarinic Acid (RA) Content

RA content in *M. × villosa* was determined using an analytical HPLC‐DAD method and system previously reported [8]. In summary, gradient elution began with 38% B, increased to 42% B at 5 min which was held until 9 min. From 9‐12 min, B increased from 42% to 45%, reaching 50% B at 15 min. The concentration was returned to 45% B at 15–17 min, held until 20 min, then returned to starting conditions of 38% B and held until 24 min stop time. Triplicate samples of extracts and standard were made at a 1:1 MeOH:acidified water (0.1% of H_3_PO_4_) dilution and filtered prior to analysis. A calibration curve was constructed using a RA standard (Sigma Aldrich) at 5, 10, 20, 40, 80 and 160 µg/ml. The calibration curve used to determine RA content and the chromatogram of MV‐OE obtained from HPLC‐DAD are available as Supporting Information, Figures S11 and S17, respectively.

### 4.5 Extractive Efficiency

Extractive efficiency (EE), defined as the ability of the solvent to extract polyphenols, was evaluated as a response, and it was calculated using Equation (5).

(5) $$\mathrm{EE}=\frac{\mathrm{TPC}}{\mathrm{DR}}\;$$

where, EE = extractive efficiency; TPC = total polyphenols content; DR = dry residue

### 4.6 Dry Residue

Dry residue (DR) was determined following the methodology described by the Brazilian regulatory agency, ANVISA [58]. For each extraction, 2 g of sample was transferred to crystallizers before drying. The solvent was evaporated using a water bath, then the crystallizers were transferred to an incubator at 100°C–105°C for 3 h to dry. Table S6, provides data for quantification of dry residue content related to factorial design samples.

### 4.7 DPPH·Radical Scavenging Activity

MV‐OE scavenging activity was evaluated according to DPPH method described by Garcez et al., 2009, with methanol as solvent. First, 5 concentrations (10, 20, 40, 60, and 80 µg/ml in triplicate) of the samples were prepared from MV‐OE stock solutions of 1 mg/ml [59]. Ascorbic acid was used as a positive control at concentrations of 5, 7.5, 10, 12.5, 15, and 17.5 µg/mL prepared from a stock solution. For each sample and control, 100 µL was transferred to a 96‐well plate along with 100 µL of 0.3 mM DPPH solution. The plates were incubated in the dark for 30 min at room temperature before measuring absorbance at 518 nm using a microplate reader (H1M, Biotek).

A negative control containing 100 µL each of methanol and DPPH was included. The scavenging activity (SA) was calculated using Equation (6). Calibration curves were constructed by plotting SA versus concentration for each sample. Results were expressed as the IC_50_ value, defined as the concentration required to inhibit DPPH radical formation by 50%. The calibration curve of ascorbic acid and MV‐OE used for DPPH assay are available as Figures S12 and S13 in Supporting Information.

(6) $$\mathrm{SA}\;\;\left(\%\right)=\left(\frac{ABS_{\mathrm{negative}\;\mathrm{control}}-ABS_{\mathrm{sample}}}{ABS_{\mathrm{negative}\;\mathrm{control}}}\right)\;\times \;100$$

### 4.8 ABTS· Radical Scavenging Activity

The ABTS radical scavenging activity was evaluated following the protocol of Re et al. (1999). Stock solutions of 7 mM ABTS and 245 mM ammonium persulfate (APS) were prepared in ultrapure water [60]. To generate the ABTS radical, 5.05 µL of the APS stock solution was added to 500 µL of the ABTS stock and incubated overnight (12–16 h) in the dark at room temperature. The ABTS radical stock solution was then diluted with ultrapure water until an absorbance of 0.700 at 734 nm was reached.

Trolox was used as a positive control and prepared as a 2 mM stock solution. Standard solutions ranging from 12.5 to 400 µM were prepared by serial dilution for a calibration curve. The test samples were prepared as 1 mg/mL stock solutions and further diluted to 100, 200, 300, 400, and 500 µg/mL.

For the assay, 10 µL of each sample or standard was transferred to a 96‐well plate along with 190 µL of the diluted ABTS radical solution. A negative control contained 10 µL ultrapure water instead of sample. The plate was incubated at room temperature in the dark for 5 min before reading the absorbance at 734 nm using a microplate reader (H1M, Biotek). All procedure was done in triplicate. The percent decolorization was calculated using Equation (7). Figures S14 and S15 of Supporting Information depicts calibration curves used to determine CE_50_ for Trolox ans MV‐OE.

(7) $$\mathrm{Deecolorization}\;\;\left(\%\right)=\left(\frac{Control\;ABS-Sample\;ABS}{Control\;ABS}\right)\times \;100$$

The antioxidant capacity of each sample was then calculated relative to the trolox calibration curve using Equation (8).

(8) $$\mathrm{Trolox}\;\mathrm{eq}\;\left(\mathrm{mg}/\mathrm{mg}\right)=\frac{Sample\;decolorization\left(\%\right)-b}{Sample\;concentration}\;$$

### 4.9 HPLC‐DAD‐ESI‐MS^n^ and HRMS Experiments

High‐performance liquid chromatography (HPLC) separation was performed using a Shimadzu system (model, Kyoto, Japan). Samples prepared with a solution of MeOH: H2O 1:1 (20 µL) were injected into a Kromasil C18 analytical column (5 µm, 100Å, 250 × 4.6 mm). The mobile phase consisted of 0.1% formic acid in water (solvent A) and methanol (solvent B).

High resolution Mass spectrometer (HRMS) experiments were performed on the HPLC system coupled with a micrOTOF II (Bruker Daltonics, Billerica, MA, USA) containing an electrospray ion (ESI) source operated in negative mode. Meanwhile, for the dereplication by tandem mass spectrometer (MS^n^) the HPLC system was coupled with an ESI Ion Trap Amazon X also in negative mode (Bruker Daltonics, Billerica, MA, USA). Dereplicated compounds were identified by comparing MS/MS fragmentation patterns to internal standards and literature data. For both mass spectrometer equipment, the acquisition parameters applied were settled as follows: capillary 4.5 kV, final plate offset 500 V, 40 psi nebulizer, dry gas (N_2_) with a flow rate of 8 mL/min and a temperature of 200°C. The mass spectra (*m*/*z* 50–1000) were recorded every 2 s. The based peak chromatogram of MV‐OE is available as Figure S16.

### 4.10 Principal Components Analysis (PCA)

LC‐HRMS spectral data files were converted to MzXML format using DataAnalysis 4.2 (Bruker). Preprocessing of the data, including feature detection, sample alignment, and peak correspondence, was performed using XCMS Online [61]. The integral values of each feature (*m/z*) were analyzed as a function of retention time. The files were then converted into CSV format. Principal component analysis (PCA) was conducted on the processed data using Unscrambler version 10.4 (CAMO Process AS, Norway) with area normalization applied to each sample.

### 4.11 In Vitro Determination of Sun Protection Factor (SPF), UVA Protection Factor (UVA‐PF), and Critical Wavelength (*λ*c)

In vitro SPF, UVA‐PF, and *λ*_c_ were determined for formulations containing MV‐OE and the chemical filter ethylhexyl methoxycinnamate (Table 4). SPF was measured using an Optometrics SPF‐290S Analyzer (Optometrics Corporation, Essex, UK). Each formulation (110 mg) was applied to Transpore tape (3 M, MN, USA) to achieve an even film with 2 mg/cm^2^ density. The samples were exposed to a 125 W CW xenon arc solar simulator, which measured the spectral transmittance (T) at 12 different spots on each tape from 290 to 400 nm in 2 nm intervals. The monochromatic protection factor was calculated at each wavelength using Equation (9). SPF and UVA‐PF values were determined using Equations (10) and (11), respectively. Critical wavelength (*λ*_c_), defined as the wavelength at which the integral of the spectral absorbance curve reaches 90% of the integral from 290 nm to 400 nm, was calculated using Equation (12). Formulations with *λ*_c_ greater than 370 nm were considered to have broad‐spectrum protection (Table 5). The results for each formulation prototype, used in photoprotective tests, are provided as Supporting Information Table S8.

**TABLE 4:** Composition of prototype formulations.

Prototype formulation	Component	Concentration (%, w/w)
1	MV‐OE Polawax cream	5% q.s.
2	MV‐OE ^a^EHMC Polawax cream	5% 1% q.s.
3	MV‐OE EHMC Polawax cream	5% 3% q.s.
4	MV‐OE Polawax cream	10% q.s.
5	MV‐OE EHMC Polawax cream	10% 1% q.s.
6	MV‐OE EHMC Polawax cream	10% 3% q.s.
7	EHMC Polawax cream	1% q.s.
8	EHMC Polawax cream	3% q.s.

^a^ Ethylhexyl metoxycinnamate.

**TABLE 5:** Wavelength (*λ*)_c_ interval and its relation to level of protection and UVA/UVB ratio.

*λ*_c_	Protection level (UVA/UVB ratio)
340 nm ≤ *λ* < 370 nm	Intermediate
*λ*_c_ > 370 nm	Broad‐spectrum

(9) $$MPF=\frac{1}{T}\;$$

(10) $$SPF=\frac{\sum_{290}^{400}E\lambda .B\lambda }{\sum_{290}^{400}\frac{E\lambda .B\lambda }{MPF\lambda }}\;$$

(11) $$UVAPF=\frac{\sum_{290}^{400}E\lambda .P\lambda }{\sum_{290}^{400}\frac{E\lambda .P\lambda }{MPF\lambda }}\;$$

(12) $$R=\frac{\int_{290\;nm}^{\lambda }A\lambda .d\lambda }{\int_{290\;nm}^{400\;nm}A\lambda .d\lambda }\;$$

where,

E = Spectral irradiance of terrestrial sunlight (under controlled conditions), B = Erythema effectiveness, MPF = Monochromatic protection factor and P = Persistent Pigment Darkening (PPD) action spectrum, A = absorbance [62].

### 4.12 Statistical Analysis

Factorial design statistical analysis was carried out, and a polynominal second‐order equation was determined by the Partial Less Square (PLS) method, using STATISTICA software 6.0 (StatSoft, USA). Statistical comparisons among groups for DPPH, ABTS, total polyphenol content, and rosmarinic acid quantification were performed using one‐way ANOVA followed by Tukey's post hoc test. Differences were considered statistically significant at *p* < 0.05. Tables S2–S5 and S7, exhibit the results ANOVA analysis for each selected parameter and a summary of the triplicate results of the samples produced to factorial design analysis, respectively.

## Author Contributions

**Juliana de M. Gomes**: conceptualization, formal analysis, investigation, methodology, writing – original draft. **Thalisson A. de Souza**: formal analysis, investigation, methodology, writing – original draf. **Márcio Vinícius. C. Terto**: data curation and investigation. **Maria Beatriz M. Nunes**: investigation and methodology. **Hariel M. U. Tesssaro**: data curation, formal analysis, investigation and software. **Luiz Alberto L. Soares**: validation, software, supervision. **Joanda Paolla R. e Silva**: data curation, formal analysis, investigation and methodology. **Samuel Cibulski**: formal analysis, investigation, visualization and writing – review & editing. **Vera Isaac**: investigation, methodology, software, Supervision. **Josean F. Tavares**: conceptualization, data curation, visualization and supervision. **Marcelo Sobral da Silva**: conceptualization, project administration, visualization, supervision.

## Conflicts of Interest

The authors declare no conflicts of interest.

## Supporting information



Supporting Information for this article is available on the WWW under https://doi.org/10.1002/cbdv.71764.
**Supporting File**: cbdv71764‐sup‐0001‐SuppMat.docx.

## Data Availability

The data that support the findings of this study are available in the Supporting Information of this article.

## References

[1] J. Lee and C.‐G. Hyun , “Natural Products for Cosmetic Applications,” Molecules 28 (2023): 534. 10.3390/molecules28020534 PMC9867114 36677594

[2] X. Tang , T. Yang , D. Yu , H. Xiong , and S. Zhang , “Current Insights and Future Perspectives of Ultraviolet Radiation (UV) Exposure: Friends and Foes to the Skin and Beyond the Skin,” Environment International 185 (2024): 108535. 10.1016/j.envint.2024.108535 38428192

[3] R. G. de Oliveira Júnior , S. A. G. B. Reis , A. P. de Oliveira , et al., “Photoprotective Potential of *Passiflora Cincinnata* Mast. (Passifloraceae) Hydro‐Alcoholic Extracts,” Chemistry and Biodiversity 21 (2024): e202401271, 10.1002/cbdv.202401271. 39087482

[4] M. B. F. M. Nunes , T. A. de Souza , J. de M Gomes , et al., “Antioxidant Potential, Photoprotective Activity, and HPLC‐ESI‐MS Profiling of Evolvulus Species From the Brazilian Caatinga,” Quimica Nova 49 (2026): 1–7, 10.21577/0100-4042.20260001.

[5] S. Tavaszi‐Sárosi , A. Sfaxi , Á. Juhász , P. Radácsi , and K. Patonay , “Chemical and Biological Properties of Mentha × villosa Huds. (mojito mint) And Its Parental Species—Mentha spicata L. (spearmint) and Mentha suaveloens Ehrh. (apple mint)—A Review,” Phytochemistry Reviews 25 (2025) 2065–2116, 10.1007/s11101-025-10136-3.

[6] C. J. L. Tenório , S. L. Silva , J. P. Raimundo e Silva , J. F. Tavares , M. R. A. Ferreira , and L. A. L. Soares , “HPLC‐ESI‐MSn Analysis and Validation of UV–Vis and RP‐HPLC‐PDA Methods for Polyphenols Quantification From Hymenaea eriogyne,” Revista Brasileira de Farmacognosia 32 (2022): 365–374.

[7] B. Cabral , T. A. F. Gonçalves , L. S. Abreu , et al., “Cardiovascular Effects Induced by Fruit Peels From Passiflora Edulis in Hypertensive Rats and Fingerprint Analysis by HPLC‐ESI‐MSn Spectrometry,” Planta Medica 88 (2022): 356–366. 10.1055/a-1385-8863 34344056

[8] J. de M Gomes , M. V. Cahino Terto , S. Golzio do Santos , M. Sobral da Silva , and J. Fechine Tavares , “Seasonal Variations of Polyphenols Content, Sun Protection Factor and Antioxidant Activity of Two Lamiaceae Species,” Pharmaceutics 13 (2021): 110. 10.3390/pharmaceutics13010110 PMC7829895 33467160

[9] I. H. Osman , “Saudi, In Vitro Antioxidant Activity of Mentha Pulegium From Arabia,” Bioscience Research 10 (2013): 33–37.

[10] O. Yumrutas and S. D. Saygideger , “Determination of Antioxidant and Antimutagenic Activities of Phlomis Armeniaca and Mentha Pulegium,” Journal of Applied Pharmaceutical Science 2 (2012): 36–40.

[11] A. Benabdallah , C. Rahmoune , M. Boumendjel , O. Aissi , and C. Messaoud , “Total Phenolic Content and Antioxidant Activity of Six Wild Mentha Species (Lamiaceae) From Northeast of Algeria,” Asian Pacific Journal of Tropical Biomedicine 6 (2016): 760–766.

[12] S. Ćavar Zeljković , J. Šišková , K. Komzáková , N. De Diego , K. Kaffková , and P. Tarkowski , “Phenolic Compounds and Biological Activity of Selected Mentha Species,” Plants 10 (2021): 550. 10.3390/plants10030550 PMC8000339 33804017

[13] M. Ranjbar , M. Kiani , and A. Nikpay , “Antioxidant and Scolicidal Activities of Four Iranian Mentha Species (Lamiaceae) in Relation to Phenolic Elements,” Journal of Herbmed Pharmacology 9 (2020): 200–208.

[14] Y. Park , S.‐A. Baek , Y. Choi , J. Kim , and S. Park , “Metabolic Profiling of Nine Mentha Species and Prediction of Their Antioxidant Properties Using Chemometrics,” Molecules 24 (2019): 258. 10.3390/molecules24020258 PMC6359624 30641968

[15] B. Nickavar , A. Alinaghi , and M. Kamalinejad , “Evaluation of the Antioxidant Properties of Five Mentha Species,” Iranian Journal of Pharmaceutical Research 7 (2008): 203–209.

[16] F. Abbou , R. Azzi , K. Ouffai , et al., “Phenolic Profile, Antioxidant, and Enzyme Inhibitory Properties of Phenolic‐rich Fractions From the Aerial Parts of Mentha Pulegium L,” South African Journal of Botany 146 (2022): 196–204.

[17] F. Z. el Hassani , “Characterization, Activities, and Ethnobotanical Uses of Mentha Species in Morocco,” Heliyon 6 (2020): e05480. 10.1016/j.heliyon.2020.e05480 PMC7689164 33294657

[18] L. Saldanha , W. Vilegas , and A. Dokkedal , “Characterization of Flavonoids and Phenolic Acids in Myrcia Bella Cambess. Using FIA‐ESI‐IT‐MSn and HPLC‐PAD‐ESI‐IT‐MS Combined With NMR,” Molecules 18 (2013): 8402–8416. 10.3390/molecules18078402 PMC6270299 23863778

[19] M. D. Catarino , A. M. S. Silva , S. C. Saraiva , A. J. F. N. Sobral , and S. M. Cardoso , “Characterization of Phenolic Constituents and Evaluation of Antioxidant Properties of Leaves and Stems of Eriocephalus africanus,” Arabian Journal of Chemistry 11 (2018): 62–69.

[20] M. Cirlini , P. Mena , M. Tassotti , et al., “Phenolic and Volatile Composition of a Dry Spearmint (Mentha spicata L.) Extract,” Molecules 21 (2016): 1007, 10.3390/molecules21081007. PMC6274304 27527127

[21] J.‐Y. Zhang , Q. Zhang , N. Li , Z.‐J. Wang , J.‐Q. Lu , and Y.‐J. Qiao , “Diagnostic Fragment‐ion‐based and Extension Strategy Coupled to DFIs Intensity Analysis for Identification of Chlorogenic Acids Isomers in Flos Lonicerae Japonicae by HPLC‐ESI‐MSn,” Talanta 104 (2013): 1–9. 10.1016/j.talanta.2012.11.012 23597880

[22] T. Hofmann , E. Nebehaj , and L. Albert , “Antioxidant Properties and Detailed Polyphenol Profiling of European Hornbeam (Carpinus betulus L.) Leaves by Multiple Antioxidant Capacity Assays and High‐performance Liquid Chromatography/Multistage Electrospray Mass Spectrometry,” Industrial Crops and Products 87 (2016): 340–348, 10.1016/j.indcrop.2016.04.037.

[23] M. N. Clifford , S. Knight , and N. Kuhnert , “Discriminating Between the Six Isomers of Dicaffeoylquinic Acid by LC‐MS n,” Journal of Agricultural and Food Chemistry 53 (2005): 3821–3832. 10.1021/jf050046h 15884803

[24] J. Kang , W. E. Price , J. Ashton , L. C. Tapsell , and S. Johnson , “Identification and Characterization of Phenolic Compounds in Hydromethanolic Extracts of Sorghum Wholegrains by LC‐ESI‐MSn,” Food Chemistry 211 (2016): 215–226. 10.1016/j.foodchem.2016.05.052 27283625

[25] I. M. Abu‐Reidah , D. Arráez‐Román , M. Al‐Nuri , I. Warad , and A. Segura‐Carretero , “Untargeted Metabolite Profiling and Phytochemical Analysis of Micromeria Fruticosa L. (Lamiaceae) leaves,” Food Chemistry 279 (2019): 128–143, 10.1016/j.foodchem.2018.11.144. 30611472

[26] C. Y. H. Djande , P. A. Steenkamp , L. A. Piater , F. Tugizimana , and I. A. Dubery , “Hordatines and Associated Precursors Dominate Metabolite Profiles of Barley (Hordeum vulgare L.) Seedlings: A Metabolomics Study of Five Cultivars,” Metabolites 12 (2022): 310. 10.3390/metabo12040310 PMC9030721 35448497

[27] D. B. Silva , I. C. C. Turatti , D. R. Gouveia , M. Ernst , S. P. Teixeira , and N. P. Lopes , “Mass Spectrometry of Flavonoid Vicenin‐2, Based Sunlight Barriers in Lychnophora Species,” Scientific Reports 4 (2015): 4309. 10.1038/srep04309 PMC3945490 24603617

[28] Z. Zhu , H. Zhang , L. Zhao , et al., “Rapid Separation and Identification of Phenolic and Diterpenoid Constituents From Radix Salvia Miltiorrhizae by High‐performance Liquid Chromatography Diode‐array Detection, Electrospray Ionization Time‐of‐flight Mass Spectrometry and Electrospray Ionization Quadrupole Ion Trap Mass Spectrometry,” Rapid Communications in Mass Spectrometry 21 (2007): 1855–1865, 10.1002/rcm.3023. 17510941

[29] A. Brito , J. Ramirez , C. Areche , B. Sepúlveda , and M. Simirgiotis , “HPLC‐UV‐MS Profiles of Phenolic Compounds and Antioxidant Activity of Fruits From Three Citrus Species Consumed in Northern Chile,” Molecules 19 (2014): 17400–17421. 10.3390/molecules191117400 PMC6271594 25356563

[30] S. Wang , L. Liu , L. Wang , Y. Hu , W. Zhang , and R. Liu , “Structural Characterization and Identification of Major Constituents in Jitai Tablets by High‐Performance Liquid Chromatography/Diode‐Array Detection Coupled With Electrospray Ionization Tandem Mass Spectrometry,” Molecules 17 (2012): 10470–10493, 10.3390/molecules170910470. PMC6268525 22945027

[31] N. Martins , L. Barros , C. Santos‐Buelga , M. Henriques , S. Silva , and I. C. F. R. Ferreira , “Evaluation of Bioactive Properties and Phenolic Compounds in Different Extracts Prepared From Salvia officinalis L,” Food Chemistry 170 (2015): 378–385. 10.1016/j.foodchem.2014.08.096 25306360

[32] P. Shi , Q. He , Y. Song , H. Qu , and Y. Cheng , “Characterization and Identification of Isomeric Flavonoid O‐diglycosides From Genus Citrus in Negative Electrospray Ionization by Ion Trap Mass Spectrometry and Time‐of‐flight Mass Spectrometry,” Analytica Chimica Acta 598 (2007): 110–118. 10.1016/j.aca.2007.07.027 17693314

[33] Y. Zhang , H. Xiong , X. Xu , et al., “Compounds Identification in Semen Cuscutae by Ultra‐High‐Performance Liquid Chromatography (UPLCs) Coupled to Electrospray Ionization Mass Spectrometry,” Molecules 23 (2018): 1199. 10.3390/molecules23051199 PMC6100538 29772791

[34] Z.‐H. Li , H. Guo , W.‐B. Xu , et al., “Rapid Identification of Flavonoid Constituents Directly From PTP1B Inhibitive Extract of Raspberry ( Rubus idaeus L.) Leaves by HPLC–ESI–QTOF–MS‐MS,” Journal of Chromatographic Science 54 (2016): 805–810. 10.1093/chromsci/bmw016 PMC4890459 26896347

[35] V. Spínola , J. Pinto , and P. C. Castilho , “Identification and Quantification of Phenolic Compounds of Selected Fruits From Madeira Island by HPLC‐DAD–ESI‐MSn and Screening for Their Antioxidant Activity,” Food Chemistry 173 (2015): 14–30. 10.1016/j.foodchem.2014.09.163 25465990

[36] G. Zeng , H. Xiao , J. Liu , and X. Liang , “Identification of Phenolic Constituents in Radix Salvia Miltiorrhizae by Liquid Chromatography/Electrospray Ionization Mass Spectrometry,” Rapid Communications in Mass Spectrometry 20 (2006): 499–506, 10.1002/rcm.2332. 16402343

[37] L. Barros , M. Dueñas , M. I. Dias , M. J. Sousa , C. Santos‐Buelga , and I. C. F. R. Ferreira , “Phenolic Profiles of Cultivated, In Vitro Cultured and Commercial Samples of Melissa officinalis L. infusions,” Food Chemistry 136 (2013): 1–8. 10.1016/j.foodchem.2012.07.107 23017385

[38] Y. Zhao , Y.‐S. Chang , and P. Chen , “Differentiation of Aurantii Fructus Immaturus From Poniciri Trifoliatae Fructus Immaturus Using Flow‐injection Mass Spectrometric (FIMS) Metabolic Fingerprinting Method Combined With Chemometrics,” Journal of Pharmaceutical and Biomedical Analysis 107 (2015): 251–257. 10.1016/j.jpba.2014.12.035 PMC4351127 25622204

[39] N. Fabre , I. Rustan , E. Hoffmann , and J. Quetin‐Leclercq , “Determination of Flavone, Flavonol, and Flavanone Aglycones by Negative Ion Liquid Chromatography Electrospray Ion Trap Mass Spectrometry,” Journal of the American Society for Mass Spectrometry 12 (2001): 707–715, 10.1016/S1044-0305(01)00226-4. 11401161

[40] I. Dimkić , M. Petrović , M. Gavrilović , et al., “New Perspectives of Purple Starthistle (Centaurea calcitrapa) Leaf Extracts: Phytochemical Analysis, Cytotoxicity and Antimicrobial Activity,” AMB Express 10 (2020): 183. 10.1186/s13568-020-01120-5 PMC7550514 33044582

[41] Z. Zuo , S. He , Y. Qiu , et al., “Salvianolic Acid A Prevents UV‐Induced Skin Damage by Inhibiting the cGAS‐STING Pathway,” International Immunopharmacology 132 (2024): 111971. 10.1016/j.intimp.2024.111971 38565040

[42] Agência Nacional de Vigilância Sanitária (ANVISA), Ministério da Saúde . “Resolução RDC no 629, de 10 de março de 2022. Dispõe sobre protetores solares e produtos multifuncionais em cosméticos e internaliza a Resolução GMC MERCOSUL no 08/2011,” Diário Oficial da União, no. 51 (2022): 125, https://anvisalegis.datalegis.net/action/ActionDatalegis.php?acao=abrirTextoAto&tipo=RDC&numeroAto=00000629&seqAto=000&valorAno=2022&orgao=RDC/DC/ANVISA/MS&codTipo=&desItem=&desItemFim=&cod_menu=1696&cod_modulo=134&pesquisa=true.

[43] T. M. Cândido , M. B. Ariede , C. A. S. de , et al., “Prospecting In Vitro Antioxidant and Photoprotective Properties of Rosmarinic Acid in a Sunscreen System Developed by QbD Containing Octyl p‐Methoxycinnamate and Bemotrizinol,” Cosmetics 9 (2022): 29.

[44] T. B. Freire , C. R. R. de Castro Lima , C. A. S. de Oliveira Pinto , L. F. Borge , A. R. Baby , and M. V. R. Velasco , “Evaluation of Interaction Between Natural Antioxidants and Chemical Sunscreens Aiming the Photoprotective Efficacy,” Journal of Thermal Analysis and Calorimetry 147 (2022): 7829–7836.

[45] G. Z. Marcon , M. C. Theisen , A. M. Castro , G. J. Radke , and F. Fumagalli , “Advances in Organic UV Filters for Sunscreens Over the Past Decade,” Photochemistry and Photobiology (2026): 1–32, 10.1111/php.70099. 41909934

[46] D. D. Peres , F. D. Sarruf , C. A. de Oliveira , M. V. R. Velasco , and A. R. Baby , “Ferulic Acid Photoprotective Properties in Association With UV Filters: Multifunctional Sunscreen With Improved SPF and UVA‐PF,” Journal of Photochemistry and Photobiology B Biology 185 (2018): 46–49. 10.1016/j.jphotobiol.2018.05.026 29864725

[47] S. González , J. Aguilera , B. Berman , et al., “Expert Recommendations on the Evaluation of Sunscreen Efficacy and the Beneficial Role of Non‐filtering Ingredients,” Frontiers in Medicine (Lausanne) 9 (2022): 790207, 10.3389/fmed.2022.790207. PMC9008233 35433750

[48] G. Tortini , P. Ziosi , E. Cesa , et al., “Criticisms in the Development of High‐Protection and Broad‐Spectrum ‘Natural/Organic’ Certifiable Sunscreen,” Cosmetics 9 (2022): 56.

[49] G. Nitulescu , D. Lupuliasa , I. Adam‐Dima , and G. M. Nitulescu , “Ultraviolet Filters for Cosmetic Applications,” Cosmetics 10 (2023): 101.

[50] R. Lavker and K. Kaidbey , “The Spectral Dependence for UVA‐Induced Cumulative Damage in Human Skin,” Journal of Investigative Dermatology 108 (1997): 17–21. 10.1111/1523-1747.ep12285613 8980280

[51] H. Zulfiqar , M. Llompart , A. Castiñeira‐Landeira , A. Duque‐Villaverde , and D. Fabbri , “Assessment of UV Filter Ethylhexyl Methoxycinnamate Photoisomerization in Aquatic Environments, Cosmetics and In Vitro Skin by (micro)Extraction GC–MS Analysis,” Microchemical Journal 222 (2026): 117149.

[52] G. Giannakopoulou , P. Pavlou , S. Papageorgiou , A. Siamidi , M. Vlachou , and A. Varvaresou , “Use of Propolis and Echinacea Extracts as Alternatives to Organic Filter in Photoprotective Formulations,” Natural Product Research 40 (2026): 3143–3152. 10.1080/14786419.2025.2456087 39853158

[53] M. D. Mota , A. N. da Boa Morte , L. C. R. C. e Silva , and F. A. Chinalia , “Sunscreen Protection Factor Enhancement Through Supplementation With Rambutan (Nephelium lappaceum L) Ethanolic Extract,” Journal of Photochemistry and Photobiology B Biology 205 (2020): 111837. 10.1016/j.jphotobiol.2020.111837 32146271

[54] Y. Li , S. Zhao , Y. Li , A. J. Ragauskas , X. Song , and K. Li , “Revealing the Relationship Between Molecular Weight of Lignin and Its Color, UV‐protecting Property,” International Journal of Biological Macromolecules 223 (2022): 1287–1296. 10.1016/j.ijbiomac.2022.11.067 36395933

[55] P. I. de S Macedo , C. A. S. de O Pinto , C. F. Hiraishi , et al., “Enhancing Photoprotection and Mitigating Ex Vivo Stratum Corneum Oxidative Stress: A Multifunctional Strategy Combining Rosmarinic Acid With UVB Filters,” Antioxidants 14 (2025): 274. 10.3390/antiox14030274 PMC11939370 40227214

[56] H. Bendová , J. Akrman , A. Krejčí , et al., “In Vitro Approaches to Evaluation of Sun Protection Factor,” Toxicology in Vitro 21 (2007): 1268–1275. 10.1016/j.tiv.2007.08.022 17945463

[57] V. L. Singleton , R. Orthofer , and R. M. Lamuela‐Raventós , “[14]Analysis of Total Phenols and Other Oxidation Substrates and Antioxidants by Means of Folin‐ciocalteu Reagent,” Methods in Enzymology 299 (1999): 152–178.

[58] Agência Nacional de Vigilância Sanitária (ANVISA), Ministério da Saúde . Farmacopeia Brasileira, 6^a^ ed., vol. I. Brasília: ANVISA, 2019, seção 5.4.2.2.2, “Determinação de resíduo seco dos extratos”, 332. https://bibliotecadigital.anvisa.gov.br/jspui/handle/anvisa/140.

[59] F. R. Garcez , W. S. Garcez , L. Hamerski , and C. H. Miguita , “Fenilpropanóides e Outros Constituintes Bioativos de Nectandra Megapotamica,” Quimica Nova 32 (2009): 407–411.

[60] R. Re , N. Pellegrini , A. Proteggente , A. Pannala , M. Yang , and C. Rice‐Evans , “Antioxidant Activity Applying an Improved ABTS Radical Cation Decolorization Assay'' *Free Radic* ,” Biologie Medicale 26 (1999): 1231–1237. 10.1016/s0891-5849(98)00315-3 10381194

[61] R. Tautenhahn , G. J. Patti , D. Rinehart , and G. Siuzdak , “XCMS Online: A Web‐based Platform to Process Untargeted Metabolomic Data,” Analytical Chemistry 84 (2012): 5035–5039. 10.1021/ac300698c PMC3703953 22533540

[62] S. Kale , P. Ghoge , A. Ansari , A. Waje , and A. Sonawane , “Formulation and In Vitro Determination of Sun Protection Factor of Nigella sativa Linn. Seed Oil Sunscreen Cream,” International Journal of PharmTech Research 2 (2010): 2194–2197.

