# Supplementary material for: Chemometric‐Assisted Extraction Optimization and HPLC‐MS Profiling of Mentha × villosa Targeting Photoprotective Activity
Source: Chem Biodivers. 2026 Oct 9;23(10):e71764. doi: 10.1002/cbdv.71764 (PMC13653112; doi:10.1002/cbdv.71764)
Supplement: Supplementary file 1 [file CBDV-23-e71764-s001.docx]

Supplementary material

**Summary**

[**Figure S1.** Pareto chart for RA content from factorial design samples.	3](#_Toc234845390)

[**Figure S2.** Contour plot corresponding to RA content from factorial design samples.	3](#_Toc234845391)

[**Figure S3.** Pareto chart for total polyphenols content from factorial design samples.	3](#_Toc234845392)

[**Figure S4.** Contour plot corresponding to total polyphenols content from factorial design samples.	4](#_Toc234845393)

[**Figure S5.** Pareto chart for dry residues content from factorial design samples.	4](#_Toc234845394)

[**Figure S6.** Contour plot corresponding to dry residues content from factorial design samples.	4](#_Toc234845395)

[**Figure S7.** Pareto chart for extraction efficiency related to factorial design samples.	5](#_Toc234845396)

[**Figure S8.** Contour plot corresponding to extraction efficiency related to factorial design samples.	5](#_Toc234845397)

[**Figure S9**. Prediction values and desirability plot for the factorial design samples.	6](#_Toc234845398)

[**Figure S10.** Gallic acid (GA) calibration curve used to determine total polyphenols content in factorial design samples and MV-OE.	7](#_Toc234845399)

[**Figure S11.** RA Calibration curve used to determine RA content in factorial design samples and MV-OE.	7](#_Toc234845400)

[**Figure S12**. Ascorbic Acid (positive control) calibration curve used to determine AA CE_50_ in DPPH assay.	7](#_Toc234845401)

[**Figure S13**. MV-OE calibration curve constructed to determine its CE_50_ in DPPH assay.	8](#_Toc234845402)

[**Figure S14.** Trolox (positive control) calibration curve constructed to determine its CE_50_ in ABTS assay.	8](#_Toc234845403)

[**Figure S15.** MV-OE calibration curve constructed to determine its CE_50_ in ABTS assay.	8](#_Toc234845404)

[**Figure S16**. Based peak chromatogram (BPC) of MV-OE evaluated by HPLC-DAD-ESI-MS.	9](#_Toc234845405)

[**Figure S17**. Quantification of RA in optimized conditions Chromatogram obtained in HPLC-DAD, retention time of RA is displayed in minutes.	9](#_Toc234845406)

[**Table S1**. Raw Matrix of Factorial design 2^2^ with central points, presenting all replicates used.	10](#_Toc234845407)

[**Table S2.** ANOVA analysis of total polyphenols content related to factorial design samples.	11](#_Toc234845408)

[**Table S3**. ANOVA analysis of RA content related to factorial design samples.	11](#_Toc234845409)

[**Table S4.** ANOVA analysis of extraction efficiency related to factorial design samples.	12](#_Toc234845410)

[**Table S5.** ANOVA analysis of dry residue content related to factorial design samples.	12](#_Toc234845411)

[**Table S6.** Quantification of Dry Residue content. Triplicate results of all seven samples produced to factorial design analysis.	13](#_Toc234845412)

[**Table S7.** Triplicate results of all seven samples produced to factorial design analysis.	14](#_Toc234845413)

[**Table S8**. Results of replicates for each formulation prototype used in photoprotective tests.	15](#_Toc234845414)

# **Figure S1.** Pareto chart for RA content from factorial design samples.

# **Figure S2.** Contour plot corresponding to RA content from factorial design samples.

# **Figure S3.** Pareto chart for total polyphenols content from factorial design samples.

# **Figure S4.** Contour plot corresponding to total polyphenols content from factorial design samples.

# **Figure S5.** Pareto chart for dry residues content from factorial design samples.

# **Figure S6.** Contour plot corresponding to dry residues content from factorial design samples.

# **Figure S7.** Pareto chart for extraction efficiency related to factorial design samples.

# **Figure S8.** Contour plot corresponding to extraction efficiency related to factorial design samples.

# **Figure S9**. Prediction values and desirability plot for the factorial design samples.

# **Figure S10.** Gallic acid (GA) calibration curve used to determine total polyphenols content in factorial design samples and MV-OE.

GA Calibration curve

| GA concentration (mg/l) | Absorbance |
| --- | --- |
| 100 | 0.492333 |
| 150 | 0.771 |
| 200 | 1.02833 |
| 250 | 1.297 |
| 300 | 1.49367 |

# **Figure S11.** RA Calibration curve used to determine RA content in factorial design samples and MV-OE.

RA Calibration Curve

| RA concentration (mg/l) | Peak area |
| --- | --- |
| 5 | 239833 |
| 10 | 546581 |
| 20 | 1052999 |
| 40 | 2113352 |
| 80 | 4207978 |
| 160 | 8450591 |

# **Figure S12**. Ascorbic Acid (positive control) calibration curve used to determine AA CE_50_ in DPPH assay.

AA Calibration Curve

| AA concentration (mg/l) | Absorbance |
| --- | --- |
| 5 | 27.8872 |
| 7.5 | 35.4963 |
| 10 | 47.5473 |
| 12.5 | 61.4137 |
| 15 | 69.8725 |
| 17.5 | 81.5759 |

# **Figure S13**. MV-OE calibration curve constructed to determine its CE_50_ in DPPH assay.

MV-OE calibration curve

| MV-OE concentration (mg/l) | Absorbance |
| --- | --- |
| 10 | 18.206 |
| 20 | 25.9217 |
| 40 | 43.5196 |
| 60 | 71.3017 |
| 80 | 88.7749 |

# **Figure S14.** Trolox (positive control) calibration curve constructed to determine its CE_50_ in ABTS assay.

Trolox Calibration Curve

| Trolox concentration (mg/l) | Absorbance |
| --- | --- |
| 0.0031 | 9.05696 |
| 0.0063 | 13.5387 |
| 0.0125 | 26.1438 |
| 0.025 | 46.6853 |
| 0.0501 | 90.1961 |

# **Figure S15.** MV-OE calibration curve constructed to determine its CE_50_ in ABTS assay.

MV-OE calibration curve

| MV-OE concentration (mg/l) | Absorbance |
| --- | --- |
| 100 | 24.7767 |
| 200 | 36.2482 |
| 300 | 46.8735 |
| 400 | 59.4264 |
| 500 | 69.7696 |

# **Figure S16**. Based peak chromatogram (BPC) of MV-OE evaluated by HPLC-DAD-ESI-MS.

# **Figure S17**. Quantification of RA in optimized conditions Chromatogram obtained in HPLC-DAD, retention time of RA is displayed in minutes.

The major peaks attributed to RA showed retention times of 17.24; 17.81 and 18. 24 min, in the replicates.

# **Table S1**. Raw Matrix of Factorial design 2^2^ with central points, presenting all replicates used.

| Design: 2**(2-0) design (Spreadsheet3 in Workbook3) | | | | | | | |
| --- | --- | --- | --- | --- | --- | --- | --- |
|  | **Replicate** | **EtOH (%)** | **D:S (m/V)** | **AR** | **TPC** | **RS** | **EE** |
| **1** | 1 | -1.00000 | -1.00000 | 8.05681896 | 28.077305 | 1.01 | 27.7993118 |
| **2** | 1 | 1.00000 | -1.00000 | 7.76414817 | 23.3964539 | 0.71 | 32.952752 |
| **3** | 1 | -1.00000 | 1.00000 | 6.41467453 | 28.1624113 | 2.835 | 9.93383116 |
| **4** | 1 | 1.00000 | 1.00000 | 11.7642959 | 20.6021277 | 1.795 | 11.4775084 |
| **5 (C)** | 1 | 0.00000 | 0.00000 | 9.40135092 | 27.5312033 | 1.63 | 16.8903088 |
| **6 (C)** | 1 | 0.00000 | 0.00000 | 9.18990173 | 27.3137133 | 1.8 | 15.1742852 |
| **7 (C)** | 1 | 0.00000 | 0.00000 | 9.0325763 | 26.81253 | 1.89 | 14.1865238 |
| **8** | 2 | -1.00000 | -1.00000 | 7.69907509 | 28.3255319 | 0.93 | 30.4575612 |
| **9** | 2 | 1.00000 | -1.00000 | 7.47160993 | 23.8503546 | 0.675 | 35.3338587 |
| **10** | 2 | -1.00000 | 1.00000 | 6.2635404 | 28.2475177 | 2.785 | 10.1427353 |
| **11** | 2 | 1.00000 | 1.00000 | 11.947958 | 20.4650118 | 1.94 | 10.5489752 |
| **12 (C)** | 2 | 0.00000 | 0.00000 | 9.55900201 | 27.5524867 | 1.685 | 16.3516241 |
| **13 (C)** | 2 | 0.00000 | 0.00000 | 9.6080913 | 27.5052033 | 1.82 | 15.1127491 |
| **14 (C)** | 2 | 0.00000 | 0.00000 | 9.3673669 | 27.62577 | 1.88 | 14.6945585 |
| **15** | 3 | -1.00000 | -1.00000 | 8.35975935 | 29.33026 | 0.94 | 31.2024043 |
| **16** | 3 | 1.00000 | -1.00000 | 7.01448898 | 23.0182033 | 0.65 | 35.4126205 |
| **17** | 3 | -1.00000 | 1.00000 | 6.12045876 | 28.5312057 | 2.63 | 10.8483672 |
| **18** | 3 | 1.00000 | 1.00000 | 11.6551017 | 20.9141844 | 1.86 | 11.2441852 |
| **19 (C)** | 3 | 0.00000 | 0.00000 | 9.72108547 | 27.24752 | 1.79 | 15.2220782 |
| **20 (C)** | 3 | 0.00000 | 0.00000 | 8.83970672 | 27.5595733 | 1.755 | 15.7034606 |
| **21 (C)** | 3 | 0.00000 | 0.00000 | 9.0134552 | 27.40118 | 1.735 | 15.7931873 |

*D:S = drug:solvente ratio; AR = rosmarinic acid; TPT = total polyphenols content; RS = dry residues; EE = extraction efficiency.

# **Table S2.** ANOVA analysis of total polyphenols content related to factorial design samples.

| ANOVA; Var.:TPT; R-sqr=.99028; Adj:.98612 (Design: 2**(2-0) design (Spreadsheet3 in Workbook3) in Fatoria_2^2_2022) 2 factors, 3 Blocks, 21 Runs; MS Pure Error=.0546221 DV: TPT | | | | | |
| --- | --- | --- | --- | --- | --- |
|  | **SS** | **df** | **MS** | **F** | **p** |
| **Blocks** | 0.3539 | 2 | 0.1769 | 3.239 | 0.111162 |
| **(1)EtOH (%)(L)** | 123.0586 | 1 | 123.0586 | 2252.909 | 0.000000 |
| **EtOH (%)(Q)** | 23.7944 | 1 | 23.7944 | 435.618 | 0.000001 |
| **(2)D:S (m/V)(L)** | 6.8640 | 1 | 6.8640 | 125.663 | 0.000030 |
| **1L by 2L** | 4.6772 | 1 | 4.6772 | 85.628 | 0.000090 |
| **Lack of Fit** | 1.2300 | 8 | 0.1537 | 2.815 | 0.112064 |
| **Pure Error** | 0.3277 | 6 | 0.0546 |  |  |
| **Total SS** | 160.3057 | 20 |  |  |  |

# **Table S3**. ANOVA analysis of RA content related to factorial design samples.

| ANOVA; Var.:AR; R-sqr=.97878; Adj:.96968 (Design: 2**(2-0) design (Spreadsheet3 in Workbook3) in Fatoria_2^2_2022) 2 factors, 3 Blocks, 21 Runs; MS Pure Error=.0894609 DV: AR | | | | | |
| --- | --- | --- | --- | --- | --- |
|  | **SS** | **df** | **MS** | **F** | **p** |
| **Blocks** | 0.11036 | 2 | 0.05518 | 0.6168 | 0.570679 |
| **(1)EtOH (%)(L)** | 18.01553 | 1 | 18.01553 | 201.3787 | 0.000008 |
| **EtOH (%)(Q)** | 4.40944 | 1 | 4.40944 | 49.2890 | 0.000417 |
| **(2)D:S (m/V)(L)** | 5.07017 | 1 | 5.07017 | 56.6747 | 0.000285 |
| **1L by 2L** | 28.31797 | 1 | 28.31797 | 316.5401 | 0.000002 |
| **Lack of Fit** | 0.67586 | 8 | 0.08448 | 0.9444 | 0.543567 |
| **Pure Error** | 0.53677 | 6 | 0.08946 |  |  |
| **Total SS** | 57.13609 | 20 |  |  |  |

# **Table S4.** ANOVA analysis of extraction efficiency related to factorial design samples.

| ANOVA; Var.:EE; R-sqr=.99189; Adj:.98841 (Design: 2**(2-0) design (Spreadsheet3 in Workbook3) in Fatoria_2^2_2022) 2 factors, 3 Blocks, 21 Runs; MS Pure Error=.9029094 DV: EE | | | | | |
| --- | --- | --- | --- | --- | --- |
|  | **SS** | **df** | **MS** | **F** | **p** |
| **Blocks** | 3.561 | 2 | 1.781 | 1.972 | 0.219647 |
| **(1)EtOH (%)(L)** | 22.924 | 1 | 22.924 | 25.389 | 0.002360 |
| **EtOH (%)(Q)** | 184.367 | 1 | 184.367 | 204.193 | 0.000007 |
| **(2)D:S (m/V)(L)** | 1385.953 | 1 | 1385.953 | 1534.985 | 0.000000 |
| **1L by 2L** | 11.789 | 1 | 11.789 | 13.057 | 0.011184 |
| **Lack of Fit** | 7.734 | 8 | 0.967 | 1.071 | 0.480385 |
| **Pure Error** | 5.417 | 6 | 0.903 |  |  |
| **Total SS** | 1621.746 | 20 |  |  |  |

# **Table S5.** ANOVA analysis of dry residue content related to factorial design samples.

| ANOVA; Var.:RS; R-sqr=.98953; Adj:.98504 (Design: 2**(2-0) design (Spreadsheet3 in Workbook3) in Fatoria_2^2_2022) 2 factors, 3 Blocks, 21 Runs; MS Pure Error=.0093944 DV: RS | | | | | |
| --- | --- | --- | --- | --- | --- |
|  | **SS** | **df** | **MS** | **F** | **p** |
| **Blocks** | 0.010674 | 2 | 0.005337 | 0.5681 | 0.594369 |
| **(1)EtOH (%)(L)** | 1.020833 | 1 | 1.020833 | 108.6635 | 0.000046 |
| **EtOH (%)(Q)** | 0.232840 | 1 | 0.232840 | 24.7848 | 0.002506 |
| **(2)D:S (m/V)(L)** | 6.645408 | 1 | 6.645408 | 707.3764 | 0.000000 |
| **1L by 2L** | 0.273008 | 1 | 0.273008 | 29.0606 | 0.001678 |
| **Lack of Fit** | 0.030215 | 8 | 0.003777 | 0.4020 | 0.883684 |
| **Pure Error** | 0.056367 | 6 | 0.009394 |  |  |
| **Total SS** | 8.269345 | 20 |  |  |  |

| **Dry Residue (DR)** | | | | | | | | |
| --- | --- | --- | --- | --- | --- | --- | --- | --- |
|  | **Experiments** | **1** | **2** | **3** | **4** | **5** | **6** | **7** |
| DR content (g) | 1 | 0.0202 | 0.0142 | 0.0567 | 0.0359 | 0.0326 | 0.036 | 0.0378 |
|  | 2 | 0.0186 | 0.0135 | 0.0557 | 0.0388 | 0.0337 | 0.0364 | 0.0376 |
|  | 3 | 0.0188 | 0.013 | 0.0526 | 0.0372 | 0.0358 | 0.0351 | 0.0347 |
|  |  |  |  |  |  |  |  |  |
|  | Mean | 0.0192 | 0.013567 | 0.055 | 0.0373 | 0.034033 | 0.035833 | 0.0367 |
|  | STDV | 0.000872 | 0.000603 | 0.002138 | 0.001453 | 0.001626 | 0.000666 | 0.001735 |
|  | Relative deviation (%) | 4.54052 | 4.443032 | 3.886829 | 3.894327 | 4.777179 | 1.858138 | 4.727344 |
|  |  |  |  |  |  |  |  |  |
|  | Dry residue (%) | 1.01 | 0.71 | 2.835 | 1.795 | 1.63 | 1.8 | 1.89 |
|  |  | 0.93 | 0.675 | 2.785 | 1.94 | 1.685 | 1.82 | 1.88 |
|  |  | 0.94 | 0.65 | 2.63 | 1.86 | 1.79 | 1.755 | 1.735 |
|  |  |  |  |  |  |  |  |  |
|  | Mean (%) | 0.96 | 0.678333 | 2.75 | 1.865 | 1.701667 | 1.791667 | 1.835 |

# **Table S6.** Quantification of Dry Residue content. Triplicate results of all seven samples produced to factorial design analysis.

|  | **Natural Variables** | | **Coded Variables** | | **Response (1)** | | | | **Response (2)** | | | | **Response (3)** | | | | | **Response variables (mean)** | | | |
| --- | --- | --- | --- | --- | --- | --- | --- | --- | --- | --- | --- | --- | --- | --- | --- | --- | --- | --- | --- | --- | --- |
| Exp. | EtOH (%) | D:S (%; m/v) | EtOH (%) | D:S (%. m/v) | RA (g%) | TPC (g%) | DR (g%) | EE | RA (%) | TPC (%) | DR (%) | EE | RA (%) | TPC (%) | DR (%) | EE | RA (g%) | | TPT (g%) | RS (g%) | EE |
| 1 | 50 | 2.5 | -1.00 | -1.00 | 8.0568 | 28.077 | 1.01 | 27.799 | 7.6991 | 28.326 | 0.93 | 30.458 | 8.3598 | 29.33 | 0.94 | 31.202 | 8.038551 | | 28.5777 | 0.96 | 29.81976 |
| 2 | 90 | 2.5 | 1.00 | -1.00 | 7.7641 | 23.396 | 0.71 | 32.953 | 7.4716 | 23.85 | 0.675 | 35.334 | 7.0145 | 23.018 | 0.65 | 35.413 | 7.416749 | | 23.42167 | 0.678333 | 34.56641 |
| 3 | 50 | 7.5 | -1.00 | 1.00 | 6.4147 | 28.162 | 2.835 | 9.9338 | 6.2635 | 28.248 | 2.785 | 10.143 | 6.1205 | 28.531 | 2.63 | 10.848 | 6.266225 | | 28.31371 | 2.75 | 10.30831 |
| 4 | 90 | 7.5 | 1.00 | 1.00 | 11.764 | 20.602 | 1.795 | 11.478 | 11.948 | 20.465 | 1.94 | 10.549 | 11.655 | 20.914 | 1.86 | 11.244 | 11.78912 | | 20.66044 | 1.865 | 11.09022 |
| 5 | 70 | 5.0 | 0.00 | 0.00 | 9.4014 | 27.531 | 1.63 | 16.89 | 9.559 | 27.552 | 1.685 | 16.352 | 9.7211 | 27.248 | 1.79 | 15.222 | 9.560479 | | 27.44374 | 1.701667 | 16.15467 |
| 6 | 70 | 5.0 | 0.00 | 0.00 | 9.1899 | 27.314 | 1.8 | 15.174 | 9.6081 | 27.505 | 1.82 | 15.113 | 8.8397 | 27.56 | 1.755 | 15.703 | 9.212567 | | 27.4595 | 1.791667 | 15.33016 |
| 7 | 70 | 5.0 | 0.00 | 0.00 | 9.0326 | 26.813 | 1.89 | 14.187 | 9.3674 | 27.626 | 1.88 | 14.695 | 9.0135 | 27.401 | 1.735 | 15.793 | 9.137799 | | 27.27983 | 1.835 | 14.89142 |

# **Table S7.** Triplicate results of all seven samples produced to factorial design analysis.

# **Table S8**. Results of replicates for each formulation prototype used in photoprotective tests.

| **Formulation** | **Triplicates** | **SPF** | **SPF**  **Mean** | **SPF**  **STDV** | **UVA**  **PF** | **UVA PF**  **Mean** | **UVA PF**  **STDV** | **λc**  **(nm)** | **λc**  **Mean** | **λc**  **STDV** |
| --- | --- | --- | --- | --- | --- | --- | --- | --- | --- | --- |
| **1** | 1 | 5.61 | 5.346 | 0.255 | 4.44 | 4.266 | 0.170 | 374.4 | 374.100 | 0.264 |
|  | 2 | 5.33 |  |  | 4.26 |  |  | 374.0 |  |  |
|  | 3 | 5.10 |  |  | 4.10 |  |  | 373.9 |  |  |
| **2** | 1 | 13.44 | 13.153 | 0.667 | 6.47 | 6.316 | 0.393 | 368.7 | 368.466 | 0.321 |
|  | 2 | 12.39 |  |  | 5.87 |  |  | 368.6 |  |  |
|  | 3 | 13.63 |  |  | 6.61 |  |  | 368.1 |  |  |
| **3** | 1 | 18.91 | 17.093 | 1.575 | 9.09 | 8.010 | 0.937 | 366.6 | 366.100 | 0.624 |
|  | 2 | 16.27 |  |  | 7.40 |  |  | 365.4 |  |  |
|  | 3 | 16.10 |  |  | 7.54 |  |  | 366.3 |  |  |
| **4** | 1 | 12.32 | 9.736 | 2.260 | 10.00 | 7.846 | 1.882 | 379.9 | 379.666 | 0.251 |
|  | 2 | 8.12 |  |  | 6.51 |  |  | 379.7 |  |  |
|  | 3 | 8.77 |  |  | 7.03 |  |  | 379.4 |  |  |
| **5** | 1 | 23.54 | 23.200 | 1.023 | 12.41 | 12.253 | 0.262 | 374.6 | 374.666 | 0.305 |
|  | 2 | 22.05 |  |  | 12.40 |  |  | 374.4 |  |  |
|  | 3 | 24.01 |  |  | 11.95 |  |  | 375.0 |  |  |
| **6** | 1 | 30.49 | 30.626 | 2.098 | 16.10 | 16.563 | 1.839 | 372.3 | 372.733 | 0.404 |
|  | 2 | 32.79 |  |  | 18.59 |  |  | 373.1 |  |  |
|  | 3 | 28.60 |  |  | 15.00 |  |  | 372.8 |  |  |
| **7** | 1 | 4.19 | 4.230 | 0.262 | 1.64 | 1.656 | 0.0568 | 330.0 | 330.233 | 0.208 |
|  | 2 | 4.51 |  |  | 1.72 |  |  | 330.4 |  |  |
|  | 3 | 3.99 |  |  | 1.61 |  |  | 330.3 |  |  |
| **8** | 1 | 5.87 | 6.266 | 0.472 | 2.13 | 2.296 | 0.181 | 331.4 | 333.266 | 1.616 |
|  | 2 | 6.79 |  |  | 2.49 |  |  | 334.2 |  |  |
|  | 3 | 6.14 |  |  | 2.27 |  |  | 334.2 |  |  |
